# Developing and Testing an Online Portal for Virtual Navigation for Asian American Patients With Cancer: Pilot Feasibility Study

**DOI:** 10.2196/69097

**Published:** 2025-10-17

**Authors:** Janet N Chu, Debora Oh, Laura Allen, Janice Y Tsoh, Katarina Wang, Mei-Chin Kuo, Ching Wong, Hoan Bui, Junlin Chen, Andrea Hwang, Carmen Ma, Angeline Truong, Feng-Ming Li, Tung T Nguyen, Scarlett L Gomez, Salma Shariff-Marco

**Affiliations:** 1 Department of Medicine, University of California, 490 Illinois Street, San Francisco, CA, 94158, United States, 1 415-514-8686; 2 Asian American Research Center on Health, Department of Medicine, San Francisco, CA, United States; 3 Department of Epidemiology and Biostatistics, University of California San Francisco, San Francisco, CA, United States; 4 Department of Psychiatry and Behavioral Sciences, University of California San Francisco, San Francisco, CA, United States

**Keywords:** Asian American patients, virtual patient navigation, online portal, cancer support, health related-quality of life

## Abstract

**Background:**

Asian American patients have reported unique needs and barriers related to cancer care. While patient navigation can facilitate care coordination and help address barriers to care, in-person navigation is time and resource intensive. Virtual patient navigation can extend the benefits of patient navigation to more patients, especially those with non-English language needs.

**Objective:**

This study aimed to develop, implement, and test an online portal providing virtual navigation, including access to resources in English, Chinese, and Vietnamese, for Asian American patients with newly diagnosed colorectal, lung, or liver cancer.

**Methods:**

The online portal was built on a secure, cloud-based platform. We recruited adults aged 21 years or older with a recent diagnosis of stage I-IV colorectal, lung, or liver cancer; who identified as Asian American; spoke English, Cantonese, Mandarin, or Vietnamese; and resided in the Greater San Francisco Bay Area, California. Participants were assigned a language-concordant navigator who assessed their needs and provided tailored resources and support over 6 months through the online portal. Participants completed baseline, 3-month, 6-month, and user experience surveys. We report descriptive statistics on sociodemographic characteristics, quality of life (Functional Assessment of Cancer Therapy–General [FACT-G]), and user experiences. We used generalized estimating equations (GEE) to analyze repeated measures of quality of life.

**Results:**

The online portal included (1) a public-facing landing page, (2) a navigator interface, and (3) a participant interface, which were all available in English, Chinese, and Vietnamese. Among 51 participants, 47 (92%) and 49 (96%) completed the 3- and 6-month surveys, respectively. The mean age was 58 (SD 13) years, with 37 (73%) men, 33 (65%) speaking English, and 20 (39%) having less than a college education. Twenty-six participants (51%) had colorectal cancer, 21 (41%) had lung cancer, and 4 (8%) had liver cancer. The average total FACT-G score was 73.0 (SD 17) at baseline, 73.2 (SD 17) at 3 months, and 75.1 (SD 19) at 6 months. In GEE models, participants reported an increase in emotional well-being at 6 months compared to baseline (coefficient 0.99, 95% CI 0.01‐1.97). Among the 47 participants who completed the user experience survey, some reported issues with registering and logging into the portal, but 44 (94%) reported that the program was culturally appropriate, 35 (74%) found calls from the navigators helpful, and 35 (74%) would recommend the program to others.

**Conclusions:**

This multilingual virtual patient navigation program for Asian American patients with cancer was deemed culturally appropriate and helpful in our pilot study. Emotional well-being improved among users of the portal. Some participants reported technical challenges, but most were satisfied with the program. Language-concordant virtual patient navigation and online supportive care tools can extend the reach and benefits of patient navigation.

## Introduction

Patient navigation is a patient-centered health care delivery strategy that aims to help patients and their families identify and overcome health care barriers [1]. Patient navigation is an effective intervention for improving access to care, facilitating coordination of care, and increasing rates of preventive cancer screening, particularly for underserved patients [1]. Importantly, patient navigation can also help address linguistic and cultural barriers that contribute to disparities in health care access and outcomes [2]. Recognizing the critical role of social drivers of health, patient navigation can provide patients with guidance and support to address barriers related to finances, transportation, housing, food access, mental health, and language [3-6]. Navigators can also provide logistical guidance in navigating the complex health care system, especially for diseases such as cancer that can involve multiple health care providers [7,8]. However, patient navigation programs are human resource–intensive and can be confined by geography or the health care system. As a result, they are not easily scalable or inclusive of broader patient populations.

Cancer is the leading cause of death among Asian American adults [9]. Asian American patients with cancer have reported barriers to care, including communication challenges and language discordance with their health care team, limited information about cancer diagnosis and treatment, and difficulty in navigating the health care system [10-12]. They have also reported unmet needs related to their physical health, psychosocial stress, finances, and overall quality of life [13]. Virtual patient navigation and online supportive care tools have the potential to extend the benefits of patient navigation to a wider audience, particularly those with non-English language preferences, who face increased barriers to obtaining care and accessing resources [14-16]. Such tools can also help address the complex medical, emotional, social, and logistical needs of patients with cancer, thereby improving quality of life, physical and emotional functioning, and reducing symptom distress [17-21].

Patient Cancer OUtreach, Navigation, Technology, and Support (COUNTS) is a pilot program designed to provide resources and support to Asian American patients with cancer and their caregivers through virtual navigation from a language-concordant patient navigator and an online portal. In the first phase, we designed, implemented, and pilot-tested an in-person patient navigation intervention, which was reported previously [22]. We refined the patient navigation program based on feedback from the in-person pilot test for the virtual navigation phase of our study. Here, we describe the design and development of an online portal to facilitate patient navigation. We then pilot-tested this online portal among participants and navigators in English, Chinese, and Vietnamese and reported on the usability. Our focus is to demonstrate how this novel approach can showcase the scalability of a language-concordant, virtual patient navigation model and improve the quality of life among Asian American patients with newly diagnosed colorectal, lung, or liver cancer.

## Methods

### Patient COUNTS Portal Design and Development

A Patient Advisory Council (PAC) was convened to guide the development and implementation of Patient COUNTS. The PAC consisted of 15 Asian American members who were patients with cancer and/or survivors, caregivers, community leaders, academic partners, and cancer clinicians. The PAC met every 2 months starting in September 2018 through June 2022.

**Figure 1.**
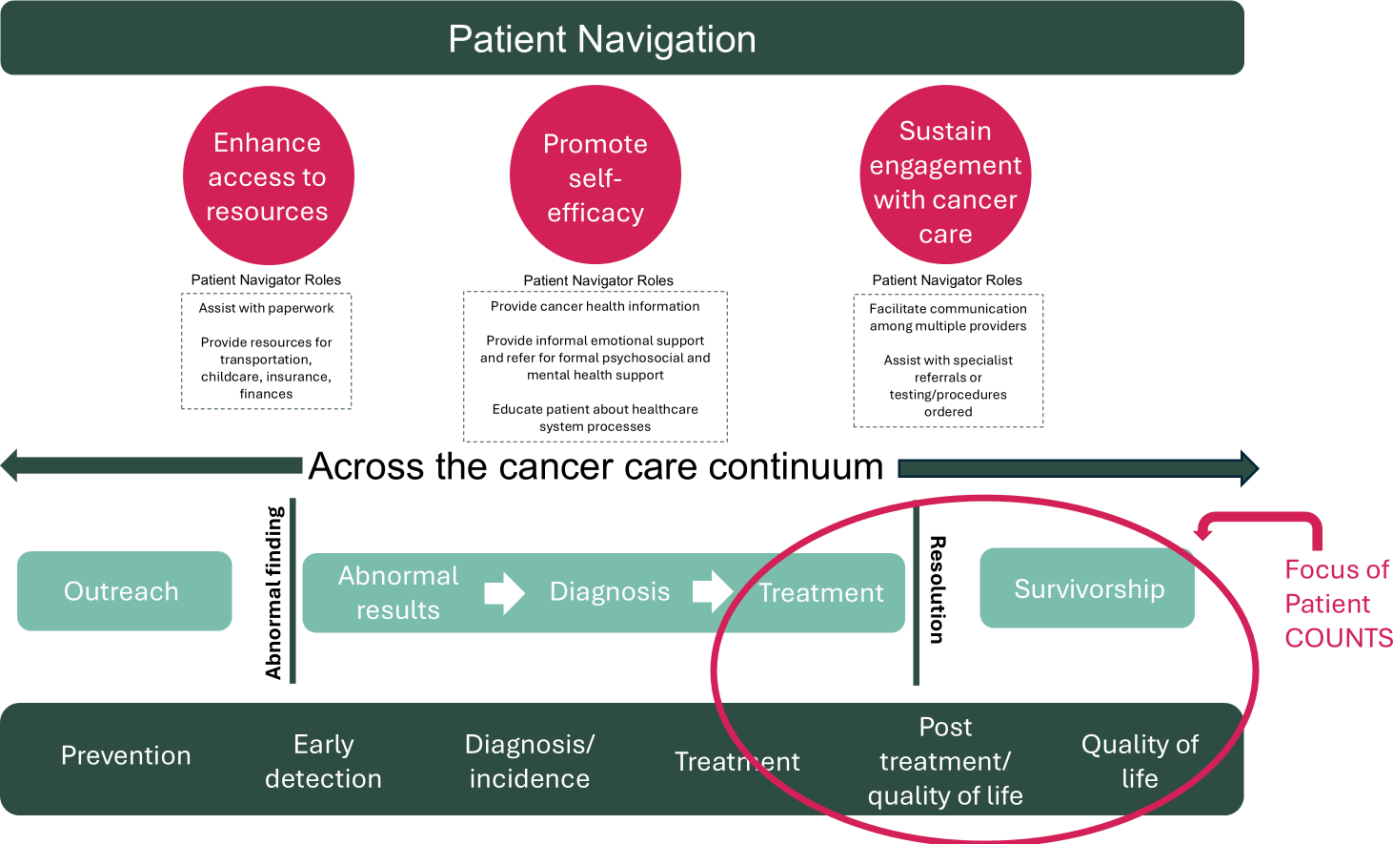
Patient navigation across the cancer care continuum framework (adapted from McKenney et al [23] and Freeman and Rodriguez [24]).

We adapted frameworks by McKenney et al [23] on patient navigator roles as well as Freeman and Rodriguez [24] on patient navigation across the health care continuum for the intervention (Figure 1). The roles of our patient navigators included helping participants enhance access to care, promote self-efficacy, and sustain engagement with care. The study team worked with the UCSF School of Medicine Technology Services (SOM Tech) to develop an online portal to facilitate virtual patient navigation for Asian American patients with cancer, with these patient navigator roles in mind.

The online patient portal was built in Salesforce, a secure, Health Insurance Portability and Accountability Act (HIPAA)–compliant, cloud-based platform, and project management was handled in Jira (Atlassian Corporation). The portal was developed using an iterative and collaborative process over two 5-month build periods. Upon completion of each of the 2 builds, the SOM Tech team met with the study team to conduct usability testing and user training, particularly for the patient navigators. Finally, the team worked closely with SOM Tech, the PAC, and the Shanti Project (a community-based organization that provides navigation to patients with cancer in San Francisco) to ensure the online portal was user-friendly, culturally relevant, and linguistically accurate. Feedback on the portal was gathered during focus groups and PAC meetings at multiple points during development. Modifications to the portal were made based on priority and budget. All elements of the portal were available in English, Chinese, and Vietnamese; translation and back-translation for the portal were conducted by multilingual staff.

The study team worked with the Shanti Project to develop a database of trusted local, tailored resources related to health care access, financial and legal issues, social and emotional support, transportation, food and nutrition, cancer information, housing, and other issues. The database was integrated into the navigator interface of the online portal so that navigators could identify appropriate resources to share with participants based on the needs reported by participants. Over the course of the study, navigators also added new resources to the database based on new needs identified by participants.

### Online Portal Implementation, Testing, and Evaluation: Participant Population and Recruitment

The implementation and testing of virtual patient navigation via the online portal for Patient COUNTS occurred from October 2020 to May 2022. Adult patients with recently diagnosed cancers within the previous 6 months were identified using an early-case ascertainment process from the Greater Bay Area Cancer Registry, a population-based cancer registry that covers 9 counties (Alameda, Contra Costa, Marin, Monterey, San Benito, Santa Clara, Santa Cruz, San Francisco, and San Mateo) in California. After receiving lists of newly diagnosed patients with cancer from the Greater Bay Area Cancer Registry, letters were sent to the physician on record to ensure that there was no medical contraindication for study participation. If there were no responses from the physician after 14 days, an invitation letter with an opt-out option was sent to the potential participant. After another 14 days, recruiters called potential participants who did not respond to the invitation letter to assess interest and eligibility. Additional recruitment approaches included informing health care providers and community organizations about the study and distributing flyers.

The eligibility criteria were: adults aged 21 years or older; self-identified as Asian American; spoke English, Cantonese, Mandarin, or Vietnamese; had stage I-IV colon, rectum, lung, or liver cancer; diagnosed in one of 9 Greater Bay Area counties; were currently receiving treatment or planning to receive treatment; and were willing to stay in the study for 6 months. Participants were excluded if they were unable to provide consent because of a medical or psychological condition or if they had already completed treatment at the time of enrollment.

Our initial goal was to enroll 50 participants in this pilot virtual patient navigation study, based on the feasibility of the available patient pool, numbers needed to assess portal effectiveness, and resource availability. We screened participants for eligibility until we reached our target sample size.

### Virtual Patient Navigation Intervention

Potential study participants accessed an eligibility screener by entering a unique code that was included in a mailed invitation letter. Upon entering their code and completing the eligibility screener, eligible participants were directed to an electronic consent form and online registration. Once registered as a study participant, participants were assigned a language-concordant patient navigator. After ensuring eligibility and obtaining consent, patient navigators connected with participants through the portal.

Navigators received training through the Shanti Project as well as supervision by study team members with expertise in issues related to Asian American patients with cancer [22]. We leveraged the pillars of our patient navigation framework to ensure the roles played by our patient navigators met the unique needs of our participants. The needs assessment survey was conducted through the online portal, where navigators assessed logistical, psychosocial, and financial needs. Based on the needs reported by each participant during their 6 months enrolled in our program, patient navigators provided logistical and emotional support. The assigned patient navigator aimed to enhance access to care by providing tailored resources to the participant through the participant’s individualized resource page on the online portal (eg, local financial and legal resources, information on where to obtain durable medical equipment, or transportation assistance). Participants could also peruse the database of local resources that our team curated on the general resource page of the online portal, which was available to all participants. Navigators promoted self-efficacy by providing emotional support or referring participants to formal psychosocial support. They also provided cancer-related health information relevant to the questions asked by participants. Finally, patient navigators encouraged sustained engagement with care by helping to schedule appointments or facilitating communication with care teams, including interpreting for patients during clinic appointments if requested.

Throughout the program, patient navigators connected with participants through the online portal, text messages, phone calls, or WeChat (Tencent Holdings Ltd), a smartphone app popular among Chinese-speaking participants. The use of multiple modes of communication ensured that navigators could reach participants using a preferred method, enhancing the overall user experience and engagement. The frequency and timing of navigator-participant interactions were tailored to the needs of each participant.

Navigators conducted baseline, 3-, and 6-month in-language surveys. These surveys could be conducted through the online portal, but were also available by phone or mail, depending on the participant’s preference.

### Survey Administration

The baseline survey and the follow-up surveys at 3 and 6 months included questions about sociodemographic characteristics; cancer type, stage, and treatment; and quality of life. Participants were also asked about needs related to patient navigation or cancer support, including financial issues, transportation or housing concerns, symptom management related to cancer or treatment side effects, mental health needs, or assistance related to language issues.

Participants who completed the 6-month program were invited to complete a user experience survey, which evaluated participants’ experiences and satisfaction with the program. If family members assisted participants with the online portal, they were also invited to complete a user experience survey. The user experience survey was administered by a trained multilingual project staff member in participants’ preferred language (English, Cantonese, Mandarin, or Vietnamese) who was not the participant’s assigned navigator and who had limited contact with the participant.

Based on participants’ preferences, all surveys (baseline, 3-month, 6-month, and user experience) were conducted online through the portal, by phone, or via mail.

### Study Measures and Variables

Sociodemographic characteristics were obtained from the baseline survey, including age, sex, highest level of education, marital status, annual household income, health insurance status, nativity, language preference, and English language fluency. Information on cancer type, stage, and treatment (including surgery, chemotherapy, or radiation) was self-reported on the baseline survey or was obtained from the cancer registry (if applicable).

We evaluated whether participants completed standard-of-care treatment based on their cancer type and stage. Standard-of-care treatment was defined from guidelines from the National Cancer Institute and reviewed by an oncologist on the team (Multimedia Appendix 1).

We used the Functional Assessment of Cancer Therapy–General (FACT-G) to assess quality of life through the baseline and both follow-up surveys. The FACT-G includes four subscales assessing (1) physical well-being, (2) social well-being, (3) emotional well-being, and (4) functional well-being. The instrument has a total of 27 items, each with a 5-point rating scale (0=not at all, 1=a little bit, 2=somewhat, 3=quite a bit, and 4=very much). For the FACT-G subscale domains in the study sample, Cronbach α values ranged from 0.72 to 0.79, suggesting acceptable internal consistency across subscales.

For this study, subscale scores were computed as the prorated sum of the item responses for that subscale as long as more than 50% of the questions comprising the subscale were answered. Prorating replaces missing values with the mean of the completed items for that subscale and is an acceptable method of imputing missing data in the FACT-G instrument [25].

The FACT-G total score was calculated as the sum of the 4 subscale scores, as long as at least 22 of the 27 items (80%) were answered; total FACT-G scores ranged from 0 to 108. Higher scores represent a better quality of life. However, due to an error in the English version of the question “I feel close to my friends,” we removed this item when calculating the social score for all participants. Therefore, the maximum total score for the FACT-G in our study was 104.

We defined clinically meaningful change as a 2-point change in a subscale domain or a 4-point change in the total FACT-G score [26].

### Data Analysis

Survey data were managed using Research Electronic Data Capture (REDCap; Paul A Harris; [27]), Qualtrics (Qualtrics^XM^), and Salesforce (Salesforce, Inc) electronic data capture tools. For survey responses, we report descriptive statistics on sociodemographic characteristics, quality of life, and user experiences. We used generalized estimating equations (GEEs) to analyze repeated measures of quality of life for total FACT-G and each subscale domain (physical well-being, social well-being, emotional well-being, and functional well-being) separately; no covariates were included in the GEE models. Stata (version 16.0; StataCorp LLC) was used to analyze the data.

### Ethical Considerations

The study was registered with the National Institutes of Health Clinical Trials Registry (NCT03867916). The University of California, San Francisco, Institutional Review Board (18‐25820) and the state of California Committee for the Protection of Human Subjects approved this study (2019‐176-UC San Francisco). All consent and study materials were available in English, Chinese, and Vietnamese. Consent was obtained by phone or online in the participant’s preferred language. For interested participants who deferred to family members, we obtained verbal consent from the participant to allow us to speak to their family member. Participants were assigned a study-specific ID number. All surveys were deidentified; only deidentified data were shared among the research team during the analysis phase. Data were stored behind the Department of Epidemiology and Biostatistics firewall on a password-protected server that was accessible only by the research team. Participants were provided a gift card of US $25 for completing each of the baseline, 3-month, 6-month, and user experience surveys (up to US $100 total).

## Results

### Portal Development

The online portal was built in 2 phases. During the first build (May to September 2019), the team built the infrastructure in Salesforce Health Cloud, integrated Qualtrics for data collection, built a participant interface for eligibility screening, consent, and enrollment, translated the website, developed notifications and reporting, and conducted user acceptability testing. The team received feedback from the PAC to improve the usability of the online portal, including increasing font size for readability and more clearly differentiating between how to contact the navigator for program or resource questions versus how to contact the study team for concerns about the study. Based on user feedback, the needs assessment was reordered to be completed before the rest of the survey. In addition, the resources provided to participants on the participant interface of the online platform were reorganized to prioritize relevance and language. During the second build (January to March 2020), the team worked to improve the navigator experience, created follow-up surveys, improved the participant interface on the online portal, translated the portal into Vietnamese, and made other improvements based on the feedback provided during PAC sessions and user acceptability testing.

Ultimately, the online portal included three main components: (1) a public-facing landing page, (2) a navigator interface, and (3) a participant interface. The landing page provided background information about the study, video testimonials from patients and caregivers, biographies of the patient navigators, and the study’s contact information. Patient navigators used the online portal for eligibility screening, obtaining consent, and administering the surveys. They also used the online portal to contact patients, assess needs, and provide resources relevant to those needs. On the participant interface, participants could log in to the online portal and either access the personalized resources provided by navigators or explore all the resources available in the database. The contact information for their assigned patient navigator was available on the online portal. The entire online portal was available in English, Chinese, and Vietnamese. During the study, 51 participants, 16 caregivers, and 4 patient navigators used the online portal.

The study team collated an extensive database of 167 local resources to provide to participants in the following domains: (1) affordable medical and dental care, as well as alternative medicine services; (2) financial, transportation, legal, or housing needs; (3) activities for patients with cancer and survivors; (4) caregiver resources for both children and adults; (5) information about side effects of treatment, as well as organizations that provided cosmetic and durable medical equipment to help mitigate some of those side effects; (6) mental health services, including individual counseling and support groups; and (7) local food resources.

### Online Portal Testing and Evaluation: Participant Recruitment and Baseline Characteristics

**Figure 2.**
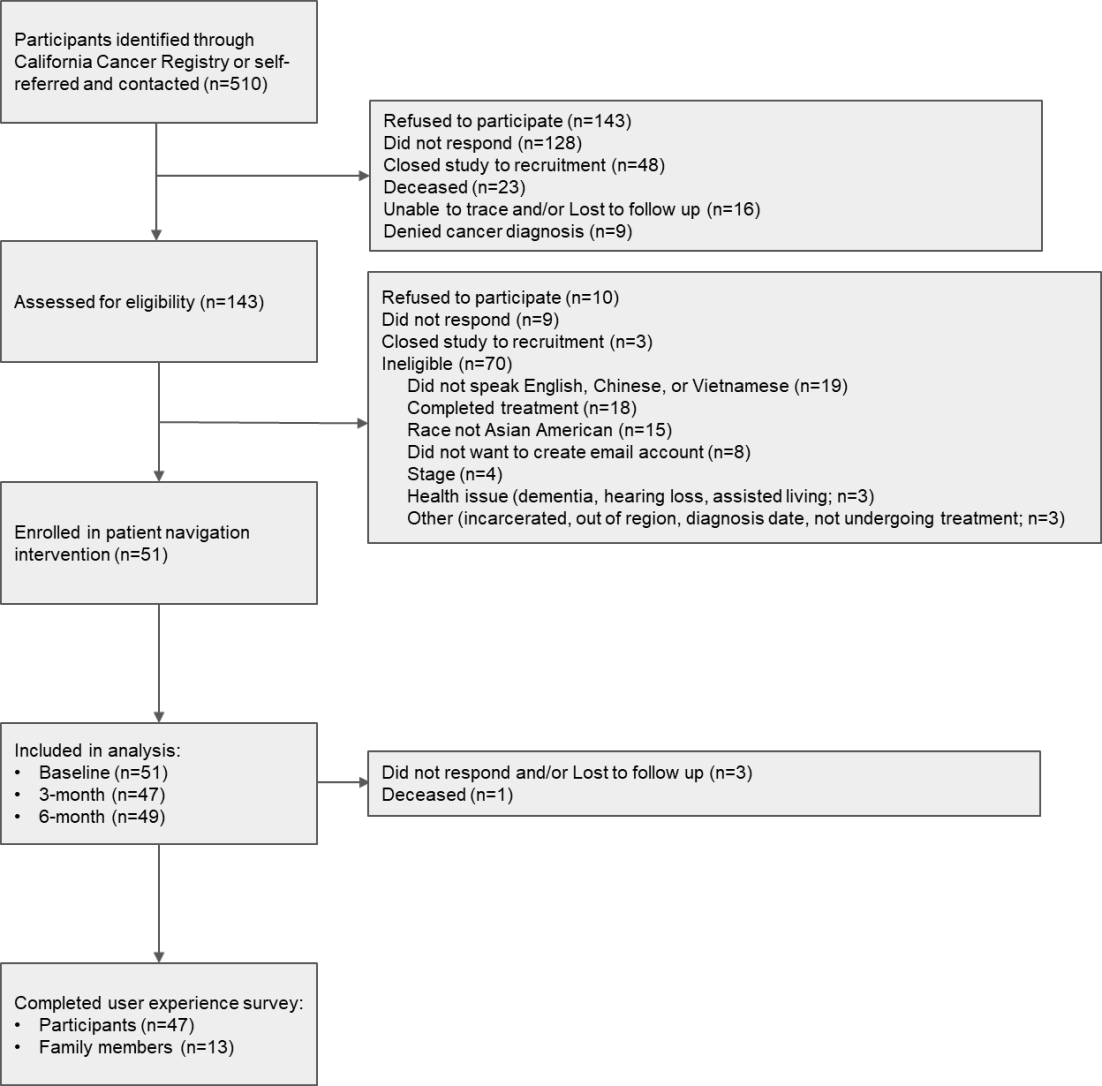
Flow diagram for participants in the Patient Cancer OUtreach, Navigation, Technology, and Support (COUNTS) pilot virtual patient navigation program.

Of 510 potential participants with newly diagnosed colorectal, lung, or liver cancer who were identified for potential eligibility, including 1 participant who self-referred, 143 (28%) refused, 128 (25%) did not respond, 23 (5%) were deceased, 16 (3%) were lost to follow-up, and 9 (2%) denied having a cancer diagnosis (Figure 1). In addition, 48 were sent letters, but did not receive follow-up phone calls for enrollment due to the study’s closure to recruitment. Among the remaining 143 participants who were assessed for eligibility, 10 (7%) refused, 9 (6%) did not respond, and 3 (2%) were not fully followed up on due to the study closing to recruitment; 70 (49%) were ineligible due to not speaking English, Chinese, or Vietnamese; having already completed treatment; not identifying as Asian American; or not wanting to create an email account (Figure 2). In total, 51 participants completed the baseline survey and were enrolled in the intervention, 47 (92%) completed the 3-month follow-up survey, and 49 (96%) completed the 6-month follow-up survey. Forty-seven (92%) participants completed surveys online, and 3 (8%) participants completed surveys over the phone. Navigators and participants mostly connected through phone calls (51 participants; range 1‐27 calls) and emails (36 participants; range 1‐10 emails). Navigators only messaged 2 participants (range 1‐5 messages) through the online portal.

For the 51 participants, the average age was 58 years (SD 13), 37 (73%) were male, and 43 (84%) were married or living with a partner (Table 1). Twenty (39%) participants had less than a college education, and 15 (29%) had a household annual income of US ≤$50,000. Participants in this study largely relied on Medicare and Medi-Cal for health insurance (34/51, 66%) (Table 1). Twenty-seven (53%) participants were born in China or Hong Kong, 5 (10%) in Vietnam, and 12 (24%) in other countries outside of the United States (including India, the Philippines, Japan, Indonesia, Laos, Malaysia, Pakistan, and Taiwan). Thirty-three (65%) participants reported their preferred languages as English, 16 (31%) preferred Cantonese, and 2 (4%) preferred Vietnamese (Table 1).

**Table 1.** Sociodemographic and health care characteristics of Asian American participants in Patient Cancer OUtreach, Navigation, Technology, and Support (COUNTS; N=51).

Characteristic	Participants
Age (years), mean (SD)	58 (13)
Sex, n (%)	
Male	37 (73)
Female	14 (27)
Highest education attained, n (%)	
≤High school graduate or equivalent	14 (27)
Some college or vocational training	6 (12)
College graduate	13 (25)
Graduate degree or higher	16 (31)
Annual household income (US $), n (%)	
<20,000	9 (18)
20,000-50,000	6 (12)
>50,000	26 (51)
Marital status, n (%)	
Married, domestic partner, or living with partner	43 (84)
Single, in a relationship but not living together, or widowed	6 (12)
Insurance^a^, n (%)	
Private	28 (55)
Medi-Cal	17 (33)
Medicare	17 (33)
Place of birth^b^, n (%)	
United States	6 (12)
China/Hong Kong	27 (53)
Vietnam	5 (10)
Other	12 (24)
Preferred language, n (%)	
English	33 (65)
Cantonese/Mandarin	16 (31)
Vietnamese	2 (4)
Limited English fluency^c^, n (%)	21 (41)
Limited health literacy^d^, n (%)	24 (47)
Cancer type, n (%)	
Colorectal	26 (51)
Lung	21 (41)
Liver	<11^e^ (8)
Cancer stage, n (%)	
Stage 1	11 (26)
Stage 2	12 (29)
Stage 3	12 (29)
Stage 4	6 (14)
Cancer treatment status (at baseline)^a^, n (%)	
Had surgery	36 (71)
Started chemotherapy	29 (57)
Started radiation	16 (31)
Not planning on having treatment	1 (2)
Completed standard-of-care treatment^f^	34 (83)

a Not mutually exclusive categories.

b Other place of birth includes India, Philippines, Japan, Indonesia, Laos, Malaysia, Pakistan, and Taiwan.

c Defined as speaking English “not well” or “not at all.”

d Defined as needing help with reading instructions, pamphlets, or other written material from doctor or pharmacy “sometimes,” “often,” or “always.”

e Redacted for cell size n≤11 if data from cancer registry.

f Defined from guidelines from the National Cancer Institute and reviewed by an oncologist on the study team.

Twenty-six (51%) participants had colorectal cancer, 21 (41%) had lung cancer, and 4 (8%) had liver cancer. Twenty-three participants (55%) had stage I or II cancer. Most participants had started cancer treatment at baseline, with 36 (71%) having undergone surgery, 29 (57%) having started chemotherapy, and 16 (31%) having started radiation (Table 1). Among participants who received treatment during the study period, 34 (83%) completed standard-of-care treatment. The remaining participants had partially completed recommended treatment, but it is unknown if treatment was fully completed, as we did not follow them past the 6-month timeframe of the study.

### Participant Quality of Life: FACT-G Scores

Among all participants who completed baseline, 3-, and 6-month surveys, the average FACT-G score was 73.0 (SD 17.0), 73.2 (SD 17.2), and 75.1 (SD 19.1), respectively (Table 2).

**Table 2.** Functional Assessment of Cancer Therapy–General (FACT-G) scores at baseline, 3-month, and 6-month surveys for Patient Cancer OUtreach, Navigation, Technology, and Support (COUNTS) participants.

FACT-G domain^a^	Baseline (n=49), mean (SD)	3 months, (n=45), mean (SD)	6 months (n=46), mean (SD)
Physical well-being	20.9 (5.9)	21.7 (5.5)	21.7 (6.4)
Social well-being	16.8 (5.5)	16.4 (5.1)	17.0 (4.9)
Emotional well-being	18.1 (4.3)	18.3 (4.2)	19.1 (4.2)
Functional well-being	17.1 (6.7)	16.7 (6.4)	17.3 (7.5)
Total FACT-G^b^ score	73.0 (17.0)	73.2 (17.2)	75.1 (19.1)

a Includes participants who completed both baseline and 6-month surveys and answered at least half of each of the subscales. Answers to the statement “I feel close to my friends” were dropped from all participants in the analysis, given an incorrect transcription in English. Therefore, the total FACT-G score for our study was out of a total of 104 points (compared to the usual 108 total points), and the social well-being subscale domain had a maximum of 24 points; the subscale domains for physical, emotional, and functional well-being remained the same (maximum 28 points each).

b FACT-G: Functional Assessment of Cancer Therapy–General.

**Figure 3.**
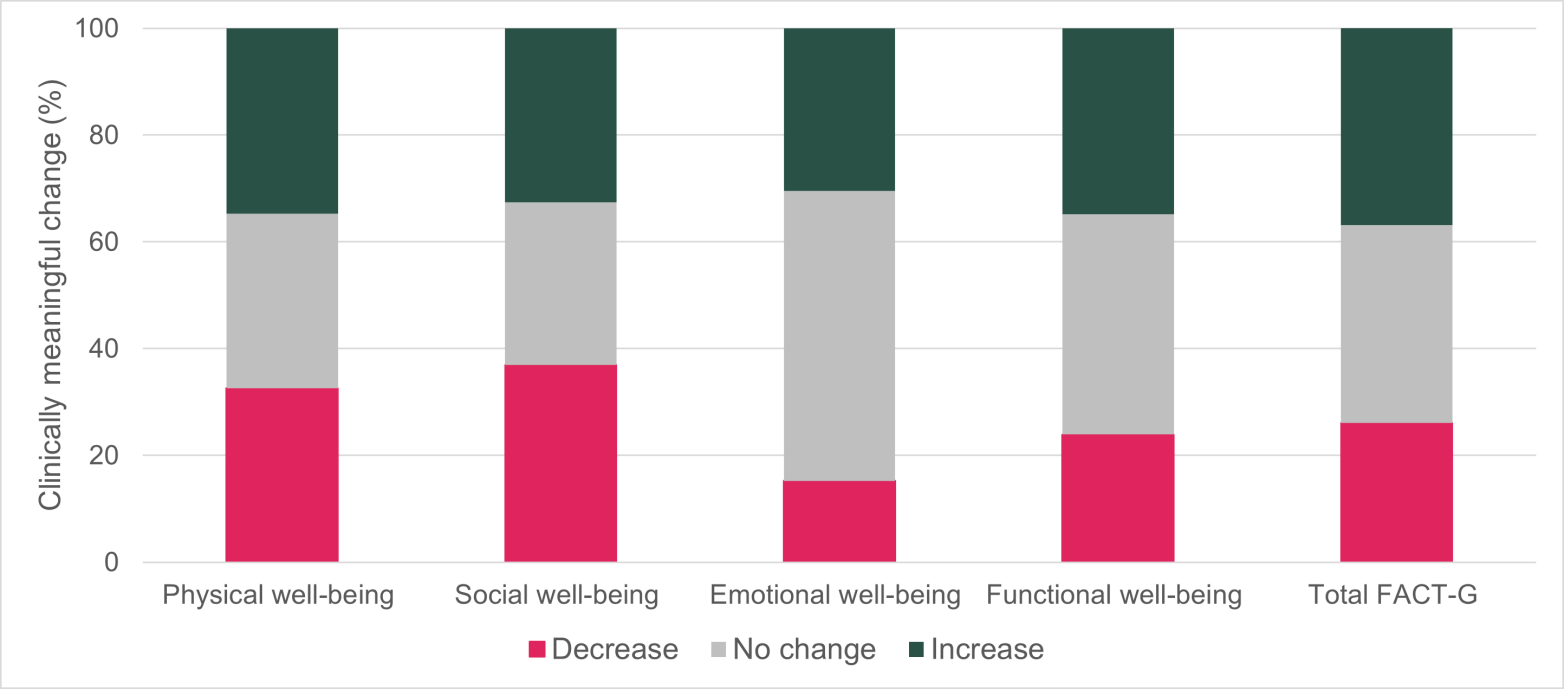
Clinically meaningful changes in Functional Assessment of Cancer Therapy–General (FACT-G) total and subscale scores from baseline to 6-month survey for Patient Cancer OUtreach, Navigation, Technology, and Support (COUNTS) participants.

Sixteen (35%) participants had a clinically meaningful increase in physical well-being, 15 (33%) in social well-being, 14 (30%) in emotional well-being, 16 (35%) in functional well-being, and 17 (37%) in total FACT-G scores (Figure 3).

Among participants who completed baseline and 6-month surveys, clinically meaningful change was defined as a 2-point change in the subscale domain or a 4-point change in total FACT-G score.

In our GEE model, only emotional well-being had a clinically meaningful increase at 6 months compared to baseline (coefficient 0.99, 95% CI 0.01‐1.97; Table 3).

**Table 3.** Generalized estimating equation (GEE) models analyzing repeated Functional Assessment of Cancer Therapy–General (FACT-G) total and subscale scores at 3 months and 6 months compared to baseline.

FACT-G^a^ domain^b^	3 months, coefficient (95% CI)	6 months, coefficient (95% CI)
Physical well-being score	0.70 (−0.67 to 2.06)	0.52 (−0.83 to 1.87)
Social well-being score	−0.55 (−1.94 to 0.84)	0.06 (−1.32 to 1.45)
Emotional well-being score	0.25 (−0.73 to 1.24)	*0.99 (0.01 to 1.97)* ^c^
Functional well-being score	−0.50 (−2.00 to 1.00)	0.17 (−1.33 to 1.66)
Total FACT-G score	−0.09 (−3.72 to 3.54)	1.74 (−1.86 to 5.34)

a FACT-G: Functional Assessment of Cancer Therapy–General.

b No covariates were included in the GEE models.

c Italicized values indicate statistically significant at *P*<.05.

### User Experience

The user experience survey was completed by 47 participants and 13 family members, representing 48 unique participants. Of 47 participants who completed the user experience survey, 44 (94%) reported that the program was culturally appropriate (“good,” “very good,” or “excellent”). In addition, 35 (74%) reported that calls from the navigator were helpful, and 37 (78%) said that navigators understood their concerns. Participants reported that the most useful features of the online portal were the needs assessment, videos describing cancer experiences among Asian American patients, and resources being available online. However, 21 (45%) participants had difficulty with the technical aspects of the portal, with 15 (32%) reporting they got help from family members to use the portal. Most participants who reported difficulty with the portal reported issues related to registering and logging in to the platform, including not knowing how to enter the code provided to them (for eligibility screening). Nevertheless, 36 (77%) participants thought the online portal was useful, 39 (83%) participants were satisfied with the overall program, and 35 (74%) participants would recommend the program to others. Among the 40 participants who received resources from a navigator, 33 (83%) said that the resources provided were helpful.

Of 13 family members who participated in the user experience survey, 9 (85%) thought calls from the navigator were helpful, and 12 (92%) said that navigators understood their concerns. Furthermore, all 13 (100%) family members thought the program was culturally appropriate (“good,” “very good,” or “excellent”). In contrast, only 4 (31%) thought the portal was useful. Overall, 9 (69%) family members were satisfied with the program, and 10 (77%) would recommend the program.

## Discussion

### Principal Findings

To our knowledge, this is the first study to develop and test a virtual patient navigation program that leveraged an online platform in multiple languages to provide support and resources to Asian American patients undergoing cancer treatment. We found that developing a virtual online patient navigation portal was technologically challenging yet feasible and successfully achieved our goal of assessing the needs of Asian American patients with cancer and connecting them with relevant resources and support. Participants reported a clinically meaningful increase in emotional quality of life between baseline and 6 months.

### Comparison to Previous Literature

Language assistance was one of the highest reported needs among participants from both phases (in-person and virtual) of Patient COUNTS [10,11]. Language discordance between patients and their clinical care teams has been associated with difficulty communicating with providers, challenges in accessing medical services, worse health status, and decreased patient satisfaction [28-32]. Building the online portal in English, Vietnamese, and Chinese and providing language-concordant patient navigation filled a critical gap in improving cancer care for Asian American patients with non-English language preference and those with limited English proficiency. In the first phase of Patient COUNTS (in-person patient navigation), 88% of participants had a non-English preferred language, and 75% reported having limited English proficiency (defined as participants who reported speaking English “not at all” or “not well”) [33]. In developing the online portal, a critical component was ensuring that the entire interface was available in Chinese, Vietnamese, and English so that participants were able to engage with the portal regardless of their language preference. In this second phase of the program, which focused on virtual patient navigation, 35% reported a non-English language preference and 41% reported limited English proficiency, which is more representative of the level of English proficiency of Asian Americans in the general US population: one-third of the Asian American population reports speaking English less than “very well” [34]. Individuals with limited English proficiency and limited health literacy also face increased challenges with digital health literacy [34]. In our study, 45% reported challenges in using the portal. Most (80%) participants who described difficulties with the portal reported issues with registering and logging in to the platform, which required an email address. Five participants reported a lack of familiarity with computer technology or challenges in navigating the portal itself. This suggests that improvements can be made to enhance the usability of our online portal and reduce barriers among populations who historically have faced increased barriers to using such online tools. Despite these challenges, the majority of participants (77%) reported that they found the online portal helpful. While our first study showed that in-person patient navigation was more attractive to participants with a non-English language preference or limited English proficiency, this study demonstrated that virtual language- and culturally concordant navigation via an online portal can effectively support a diverse sample of Asian American participants with cancer undergoing treatment.

Ensuring that patients have access to linguistically and culturally sensitive resources, particularly during vulnerable times such as with a new cancer diagnosis and while undergoing cancer treatment, is essential to improving patient satisfaction and outcomes, especially in underserved populations. Overall, the Patient COUNTS program was well received by participants and their caregivers, with high satisfaction rates. This was similar to our in-person pilot as well as other in-person cancer-related patient navigation programs [35-37]. The majority of participants found the Patient COUNTS portal useful and would recommend it to others. More participants in the in-person patient navigation program reported that their patient navigator understood their health concerns (80%), compared to about half of the participants who completed the virtual navigation. This highlights the importance of the human connection in patient navigation that contributes to patient satisfaction with the program. In particular, this is true among family members of participants, most of whom found the patient navigators to be helpful, but only one-third thought the online portal was useful. Our study found that emotional well-being improved at 6 months compared to baseline; it is possible that our patient navigators were able to contribute to participants’ emotional well-being by providing support in addition to tailored resources for our participants. About 75% of participants in our study felt that calls from their navigator were helpful and that navigators understood their concerns. In future iterations, the online portal could also include resources for family members and caregivers of participants, as many of them assist participants in accessing and navigating the health care system.

### Strengths and Limitations

Our study had several limitations. First, the study included participants recruited from the Greater San Francisco Bay Area who spoke English, Chinese, or Vietnamese; were recently diagnosed with colorectal, lung, or liver cancer; and were undergoing or planning to undergo treatment; thus, the findings may not be generalizable to other populations or geographies. However, we focused on this population to build upon previous work that found a need for navigation to address local disparities in cancer-related care. Second, longer-term experience with the portal and its impact on quality of life in a larger follow-up study would help validate the usefulness of the portal. Third, many participants reported challenges with registering and logging into the online portal. It is possible that negative first experiences with the portal may have discouraged participants from using it. To help mitigate this, patient navigators were able to walk participants through the process of registering and logging in. Fourth, while we informally asked patient navigators about their experience with the training, online portal, and intervention, future studies should obtain more formal feedback from patient navigators to help us understand the usability of the online portal and better inform future improvements. Fifth, given our small sample size, we may have lacked adequate power to detect changes in quality of life in GEE analyses; our GEE analyses were also unadjusted for potential confounders. Yet, we were able to demonstrate the feasibility of a linguistically concordant and culturally appropriate virtual patient navigation program. Finally, a lack of curated and up-to-date culturally and linguistically appropriate resources meant that our team and navigators had to manually research and confirm helpful resources for the patients.

### Lessons Learned

Our team learned several important lessons from designing, building, and implementing Patient COUNTS: (1) participants value human connection, (2) registration should be simple, (3) resources are difficult to keep up to date, and (4) building technology-enabled tools is costly. We believe that the primary reason our virtual intervention was well received was that our navigators were able to connect with participants, listen to them, and respond to their needs with a culturally sensitive and linguistically concordant approach. With respect to issues related to registration and logging in, we recommend that future tools use phone numbers with 2-factor identification for registration and login to eliminate the use of email and passwords. In addition, partnering with organizations that have regularly updated nationwide databases can address challenges with resources becoming outdated. Finally, using existing, industry-standard tools such as REDCap [27] for conducting eligibility screening and participant surveys can allow teams to spend limited financial, time, and personnel resources on the intervention rather than building these research components internally. We have incorporated these lessons into our current expansion of Patient COUNTS to racially and ethnically diverse patients with breast cancer who speak English, Spanish, or Chinese.

### Conclusion

In summary, an online portal providing virtual patient navigation to Asian American patients with cancer was challenging, yet feasible, acceptable, and helped provide linguistically and culturally sensitive support and resources. To our knowledge, this is the first virtual patient navigation program offered in multiple languages for Asian American patients undergoing cancer treatment. While improvements to the underlying platform can be made to improve the experience for participants, their caregivers, and navigators, virtual patient navigation has a high potential to reach more patients, improve health outcomes, and reduce health disparities in underserved populations.

## Supplementary material

10.2196/69097 Multimedia Appendix 1 Definition of standard of care for cancer treatment in a virtual patient navigation program for Asian American patients with cancer.

## Abbreviations:

COUNTS: Cancer OUtreach, Navigation, Technology, and Support
FACT-G: Functional Assessment of Cancer Therapy–General
GEE: generalized estimating equation
HIPAA: Health Insurance Portability and Accountability Act
PAC: Patient Advisory Council
REDCap: Research Electronic Data Capture

## References

[1] Chan RJ, Milch VE, Crawford-Williams F (2023). Patient navigation across the cancer care continuum: an overview of systematic reviews and emerging literature. CA Cancer J Clin.

[2] Natale-Pereira A, Enard KR, Nevarez L, Jones LA (2011). The role of patient navigators in eliminating health disparities. Cancer.

[3] Chu JN, Canchola AJ, Keegan THM (2022). Evaluating the impact of social and built environments on health-related quality of life among cancer survivors. Cancer Epidemiol Biomarkers Prev.

[4] Wang JH yu, Adams IF, Tucker-Seeley R (2013). A mixed method exploration of survivorship among Chinese American and non-Hispanic White breast cancer survivors: the role of socioeconomic well-being. Qual Life Res.

[5] McDaniel JT, Nuhu K, Ruiz J, Alorbi G (2019). Social determinants of cancer incidence and mortality around the world: an ecological study. Glob Health Promot.

[6] Freund KM, Battaglia TA, Calhoun E (2008). National Cancer Institute Patient Navigation Research Program: methods, protocol, and measures. Cancer.

[7] Dwyer AJ, Wender RC, Weltzien ES (2022). Collective pursuit for equity in cancer care: the National Navigation Roundtable. Cancer.

[8] Wells KJ, Campbell K, Kumar A, Clark T, Jean-Pierre P (2018). Effects of patient navigation on satisfaction with cancer care: a systematic review and meta-analysis. Support Care Cancer.

[9] Torre LA, Sauer AMG, Chen MS, Kagawa-Singer M, Jemal A, Siegel RL (2016). Cancer statistics for Asian Americans, Native Hawaiians, and Pacific Islanders, 2016: converging incidence in males and females. CA Cancer J Clin.

[10] Wang K, Ma C, Li FM (2022). Patient-reported supportive care needs among Asian American cancer patients. Support Care Cancer.

[11] Wang K, Chu JN, Oh DL (2024). Correlates of supportive care needs among Asian Americans with colorectal, liver, or lung cancer from a web-based patient navigation portal intervention: the Patient COUNTS study. Cancer Rep (Hoboken).

[12] Tan NQP, Shin LJ, Maki KG, Geng Y, Volk RJ, Lu Q (2024). A systematic review of the impact of cancer survivorship interventions with Asian American cancer survivors. Asian Am J Psychol.

[13] Chen MS (2005). Cancer health disparities among Asian Americans: what we do and what we need to do. Cancer.

[14] Shi L, Lebrun LA, Tsai J (2009). The influence of English proficiency on access to care. Ethn Health.

[15] Pandey M, Maina RG, Amoyaw J (2021). Impacts of English language proficiency on healthcare access, use, and outcomes among immigrants: a qualitative study. BMC Health Serv Res.

[16] Xie Z, Chen G, Suk R, Dixon B, Jo A, Hong YR (2023). Limited English proficiency and screening for cervical, breast, and colorectal cancers among Asian American adults. J Racial Ethn Health Disparities.

[17] Ryhänen AM, Rankinen S, Siekkinen M, Saarinen M, Korvenranta H, Leino-Kilpi H (2012). The impact of an empowering internet-based Breast Cancer Patient Pathway program on breast cancer patients’ knowledge: a randomised control trial. Patient Educ Couns.

[18] Zhang X, Xiao H (2018). Development and evaluation of a WeChat-based life review programme for patients with cancer: protocol for a randomised controlled trial. BMJ Open.

[19] Lee HY, Koopmeiners JS, Rhee TG, Raveis VH, Ahluwalia JS (2014). Mobile phone text messaging intervention for cervical cancer screening: changes in knowledge and behavior pre-post intervention. J Med Internet Res.

[20] Wang Y, Lin Y, Chen J, Wang C, Hu R, Wu Y (2020). Effects of Internet-based psycho-educational interventions on mental health and quality of life among cancer patients: a systematic review and meta-analysis. Support Care Cancer.

[21] Huang CC, Kuo HP, Lin YE, Chen SC (2019). Effects of a web-based health education program on quality of life and symptom distress of initially diagnosed advanced non-small cell lung cancer patients: a randomized controlled trial. J Canc Educ.

[22] Chu JN, Tsoh JY, Shariff-Marco S (2024). Patient COUNTS: a pilot navigation program for Asian American cancer patients. Asian Am J Psychol.

[23] McKenney KM, Martinez NG, Yee LM (2018). Patient navigation across the spectrum of women’s health care in the United States. Am J Obstet Gynecol.

[24] Freeman HP, Rodriguez RL (2011). History and principles of patient navigation. Cancer.

[25] Fairclough DL, Cella DF (1996). Functional Assessment of Cancer Therapy (FACT-G): non-response to individual questions. Qual Life Res.

[26] Cella D, Hahn EA, Dineen K (2002). Meaningful change in cancer-specific quality of life scores: differences between improvement and worsening. Qual Life Res.

[27] Harris PA, Taylor R, Thielke R, Payne J, Gonzalez N, Conde JG (2009). Research electronic data capture (REDCap)--a metadata-driven methodology and workflow process for providing translational research informatics support. J Biomed Inform.

[28] Kim G, Worley CB, Allen RS (2011). Vulnerability of older Latino and Asian immigrants with limited English proficiency. J Am Geriatr Soc.

[29] Nguyen P, Schiaffino MK, Lipton BJ (2021). Disparities in self-management outcomes by limited English proficiency among adults with heart disease. Prev Med Rep.

[30] Chu JN, Sarkar U, Rivadeneira NA, Hiatt RA, Khoong EC (2022). Impact of language preference and health literacy on health information-seeking experiences among a low-income, multilingual cohort. Patient Educ Couns.

[31] Divi C, Koss RG, Schmaltz SP, Loeb JM (2007). Language proficiency and adverse events in US hospitals: a pilot study. Int J Qual Health Care.

[32] Chu JN, Wong J, Bardach NS (2024). Association between language discordance and unplanned hospital readmissions or emergency department revisits: a systematic review and meta-analysis. BMJ Qual Saf.

[33] Pandya C, McHugh M, Batalova J (2011). Limited English proficient individuals in the United States: number, share, growth, and linguistic diversity. Migration Policy Institute.

[34] Budiman A, Ruiz NG (2021). Key facts about Asian Americans, a diverse and growing population. Pew Research Center.

[35] Smith B, Magnani JW (2019). New technologies, new disparities: the intersection of electronic health and digital health literacy. Int J Cardiol.

[36] Braun KL, Thomas WL, Domingo JLB (2015). Reducing cancer screening disparities in medicare beneficiaries through cancer patient navigation. J Am Geriatr Soc.

[37] Thai CL, Ong G, Tran T, Le Y (2022). Assessing the impact of a Patient Navigator Intervention Program for Vietnamese-American women with abnormal mammograms. J Cancer Educ.

