# Supplementary material for: Developing and Testing an Online Portal for Virtual Navigation for Asian American Patients With Cancer: Pilot Feasibility Study
Source: JMIR Cancer. 2025 Oct 17;11:e69097. doi: 10.2196/69097 (PMC12533977; doi:10.2196/69097)
Supplement: Multimedia Appendix 1 [file cancer-v11-e69097-s001.docx]

Supplemental Table 1. Definition of standard of care for cancer treatment in a virtual patient navigation program for Asian American patients with cancer

| **Lung cancer** | | | | |
| --- | --- | --- | --- | --- |
| **Small cell lung cancer** | | | | |
| Limited Disease | Extensive Disease | Recurrent Disease |  |  |
| -Surgery followed by chemotherapy or chemoradiation  -Chemotherapy and radiation  -Systemic therapy alone  -Prophylactic cranial irradiation in select cases | -Systemic therapy (chemotherapy and/or immunotherapy)  -Thoracic radiation in select cases  -Brain radiation in select cases | -Systemic therapy (chemotherapy and/or immunotherapy)  -Palliative radiation to symptomatic lesions |  |  |
| **Non-small cell lung cancer** | | | | |
| Stage 0 | Stages IA | Stages IB, IIA, and IIB | Stage IIIA/B/C | Stage IV, newly diagnosed, progressive, recurrent |
| -Surgery  -Endobronchial therapies  -Surveillance | -Surgery  -Radiation | -Surgery with adjuvant chemotherapy and/or radiation depending on margins and high-risk features  -Radiation | -Multimodality definitive therapy (surgery, systemic therapy, and/or radiation; order of treatments is individualized)  -Systemic therapy (chemotherapy and/or immunotherapy or targeted therapy) if not a candidate for definitive therapy  chemotherapy and radiation therapy  -Radiation therapy alone if not a candidate for definitive therapy | -If actionable mutation, targeted therapy for specific mutation  -Systemic therapy (chemotherapy and/or immunotherapy)  -Palliative radiation to symptomatic lesions (e.g., bone)  -Palliative gamma knife or whole brain radiation if brain metastasis  -In select oligometastatic cases, treated with definitive therapy (e.g., surgery, definitive radiation) |
| **Colorectal Cancer** | | | | |
| Stage 0 | Stage I | Stage II | Stage III | Stage IV and Recurrent |
| -Surgery | -Surgery | -Surgery  -Chemotherapy considered in only selected cases | -Surgery  -Adjuvant chemotherapy | -Palliative chemotherapy and targeted therapy  -Surgery in selected cases, such as local recurrence,  -Patients with solitary liver metastasis, may also consider surgery, neoadjuvant chemo, local ablation, adjuvant chemotherapy, intra-arterial chemo (TACE is not standard for solitary liver metastasis) |
| **Liver cancer** | | | | |
| Resectable disease | Unrespectable but amenable to either transplant/ locoregional therapy | Recurrent disease | Unresectable not amenable to locoregional therapy or disease beyond liver |  |
| -Surgical resection if resectable | -Transplant  -Radiofrequency ablation  -Transarterial embolization | -Liver transplantation  -Surgical resection  -Ablation/TACE if amenable to locoregional therapy | Palliative systemic therapy |  |

Abbreviations: TACE= Transcatheter arterial chemoembolization
